# Amorphous Elastomeric Ultra-High Molar Mass Polypropylene in High Yield by Half-Titanocene Catalysts

**DOI:** 10.3390/polym16040512

**Published:** 2024-02-14

**Authors:** Simona Losio, Fabio Bertini, Adriano Vignali, Taiga Fujioka, Kotohiro Nomura, Incoronata Tritto

**Affiliations:** 1 Institute for Chemical Sciences and Technologies “G. Natta” National Research Council, Via A. Corti 12, 20133 Milan, Italy; simona.losio@scitec.cnr.it (S.L.); fabio.bertini@scitec.cnr.it (F.B.); adriano.vignali@scitec.cnr.it (A.V.); 2 Department of Chemistry, Tokyo Metropolitan University, Tokyo 192-0397, Japanktnomura@tmu.ac.jp (K.N.)

**Keywords:** half-titanocene catalyst, atactic polypropylene, UHMWPP, thermoplastic elastomer

## Abstract

Propylene polymerizations with different ketimide-modified half-titanocene catalysts, Cp’TiCl_2_(N=C*t*Bu_2_) [Cp’ = C_5_H_5_ (**1**), C_5_Me_5_ (**2**), Me_3_SiC_5_H_4_ (**3**)], with MAO as a cocatalyst, were investigated. The obtained polymers were studied in detail by determining their microstructure, molar masses, thermal, and mechanical properties. The Cp*-ketimide, (C_5_Me_5_)TiCl_2_(N=C*t*Bu_2_) (**2**), exhibited higher catalytic activities than Cp’TiCl_2_(N=C*t*Bu_2_) (**1**,**3**), yielding higher molar mass polymers, *M*_w_ up to 1400 Kg/mol. All the synthesized polypropylenes (PP) are atactic and highly regioregular, with predominant *rrrr* pentads, especially PP prepared with catalyst **1**. Differential scanning calorimetry (DSC) established that the polymers are fully amorphous aPP, and no melting endotherm events are detected. Glass transition temperatures were detected between −2 and 2 °C. These polypropylenes have been established to be high-performance thermoplastic elastomers endowed with remarkably high ductility, and a tensile strain at break higher than 2000%.

## 1. Introduction

Polyolefins are important commercial synthetic polymers, which are predominantly synthesized by transition metal-catalyzed olefin polymerization technology. 

The discovery of homogeneous *ansa* metallocene precatalysts for stereospecific olefin polymerization is thought of as a breakthrough in this leading industrial process [1,2,3,4,5]. They allow the production of polyolefins in a much broader variety of compositions and microstructures than any other previous catalytic systems, by virtue of the notable range of ligands which differ in the steric and electronic properties.

Regarding propylene polymerization, both highly isotactic [6] and highly syndiotactic [7] polymers have been synthesized, as well as almost all microstructures in between [1,2,3,4,5,6,7,8,9,10,11]. Moreover, differences in the stereo- and regioregularity of propylene units allow for the fine adjustment of polymer microstructure and properties. 

The control of polymer molar mass at industrially practical polymerization temperatures is also central to producing poly(α-olefins) with desired properties. Variations in the cyclopentadienyl ligand solved this essential question in the cases of isotactic [11] and syndiotactic [7] polypropylenes, while there are only a restricted number of direct, high-yield processes capable of synthesizing atactic polypropylene (aPP) with high molar masses [9,10]. It is probable that the high molar mass has limited the investigation of its properties. However, high molar masses and an absence of crystallinity have a positive effect on the elastic and optical properties of aPP [9,10]. Thus, the synthesis of amorphous, high molar mass aPP is still considered a very attractive goal. 

Progress in bridged and nonbridged half-metallocenes [12,13,14,15], and so-called non-metallocene type [16,17,18,19,20] catalysts, offers several new possibilities for the purpose [21,22].

In particular, Nomura developed a class of nonbridged cyclopentadienyltitanium aryloxide and ketimide complexes [14] as high performance catalysts for the copolymerization of ethylene with α-olefins to achieve high molar mass copolymers, for high α-olefin homopolymerization, and for the copolymerization of cyclic olefin with high α-olefin or ethylene to produce copolymers with very interesting properties in high yields. No studies for the synthesis of high molar mass polypropylene with this type of catalyst have been reported so far.

Recently, ketimide-modified half-titanocenes, Cp’TiCl_2_(N=C*t*Bu_2_), showed high catalytic activity with efficient norbornene (N) incorporation in the terpolymerization of N with ethylene and α-olefins affording high molar mass polymers with uniform molar mass distribution and compositions [23]. Very recently, propylene copolymers with a series of cyclic olefins with interesting thermal properties were obtained with aryloxide complexes, such as Cp*Ti(O-2,6-*i*PrC_6_H_3_)Cl_2_ in the presence of MAO [24].

In a study of terpolymerization of ethylene and norbornene with 1-octene by Cp*Ti(O-2,6-*i*PrC_6_H_3_)Cl_2_ and Cp’TiCl_2_(N=C*t*Bu_2_) the ketimide complexes exhibited higher catalytic activities and yielded higher molar mass terpolymers, poly(E-*ter*-N-*ter-O*)s, than the aryloxo-modified ones [25].

**Scheme 1:**
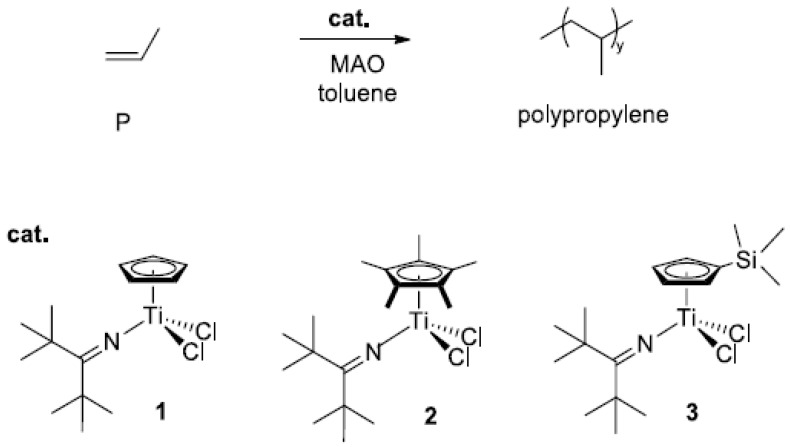
Half-titanocene-based catalysts precursors studied in propylene homopolymerization at different pressures (2 and 4 bar) and temperatures (40 and 60 °C).

Thus, we thought to explore propylene homopolymerization with these half-titanocenes. In particular, Cp’TiCl_2_(N=C*t*Bu_2_) were selected as those that gave the highest activity and molar masses in poly(E-*ter*-N-*ter*-O) [25]. Polymerization reactions were investigated by using the half-titanocene based precursors shown in Scheme 1 activated with methylaluminoxane (MAO). The influence of polymerization pressure (2 and 4 bar) and temperature (40 and 60 °C) on polymerization behavior were investigated. Polymer analysis, which includes the NMR analysis of microstructure and chain end groups and the determination of molar mass, thermal and mechanical properties, was performed. The production of ultra-high molar mass amorphous atactic PP (aUHMWPP) with excellent mechanical properties was shown.

## 2. Materials and Methods

### 2.1. Materials

All manipulations were carried out using standard Schlenk-line and glove box techniques. Propylene and nitrogen were purified by passage through column of BTS-catalysts and molecular sieves. Toluene was dried by distillation from sodium under nitrogen atmosphere prior to use. Methylaluminoxane (MAO, 10 wt% solution in toluene, Merck KGaA (Darmstadt, Germany)) was prepared by removing solvent and unreacted trimethylaluminum (50 °C, 3 h, 0.1 mm Hg) and then was stored under nitrogen. CpTiCl_2_(N=C*t*Bu_2_) (**1**) and Cp*TiCl_2_N=C*t*Bu_2_ (**2**) were synthesized according to reference [26]. C_2_D_2_Cl_4_ was purchased from Merck KGaA and used as received. 

### 2.2. Analytical Measurements

Nuclear Magnetic Resonance spectra (^1^H- and ^13^C-) were recorded on a Bruker (Billerica, MA, USA) NMR Advance 400 instrument (^13^C: pulse angle = 12.50 µs, acquisition time = 0.94 s, delay = 16 s) in C_2_D_2_Cl_4_ solution at 103 °C. For NMR analysis, about 100 mg of homopolymer was dissolved with 2 cm^3^ of deuterated solvent in a 10 mm NMR tube. Chemical shifts for ^1^H were referred to internal solvent resonances (5.86 ppm) and chemical shifts for ^13^C to internal hexamethyldisiloxane (HMDS) [δ(HMDS) -δ(TMS)] ≈ 2.0 ppm.

Differential scanning calorimetry (DSC) measurements were performed on a Perkin-Elmer (Waltham, MA, USA) DSC 8000 instrument equipped with a liquid nitrogen device. The scans were carried out from 50 to 200 °C under nitrogen atmosphere using heating and cooling rates of 20 °C/min. Glass transition temperature (*T*_g_) values were recorded during a second heating scan. Molar masses (*M*_n_ and *M*_w_) and molar mass distribution (*M*_w_/*M*_n_) were determined by high temperature size exclusion chromatography (SEC) using a Waters (Milford, MA, USA) GPCV2000 system. Measurements were carried out in *o*-dichlorobenzene at 145 °C, a polystyrene standard calibration in the 162–5.6 × 10^6^ g/mol range was used. 

The samples for the mechanical analysis were molded in a heated press at 180 °C and 50 bar for 5 min, afterwards the cooling the press plates were performed at 20 °C/min to room temperature, obtaining films with a thickness of approximately 200 µm. Dog-bone-shaped specimens (length overall 75 mm, gauge length 25 mm, and width of narrow section 4 mm) were tested at room temperature using a Zwick Roell (Ulm, Germany) ProLine Z010 dynamometer equipped with a XforceP (50 N) load cell at a crosshead speed of 15 mm/min. In the hysteresis tests, conducted at the fixed strain of 300%, the specimens were loaded and unloaded in uniaxial stretching for 10 cycles. The strain recovery (SR) was determined as SR = 100(ε_a_ − ε_r_)/ε_a_, where ε_a_ is the applied strain and ε_r_ is the strain in the cycle at zero load following the applied strain. For each material, at least five specimens were tested for tensile tests and two specimens for recovery experiments.

### 2.3. Synthesis of (Me_3_SiC_5_H_4_)TiCl_2_(N=CtBu_2_) *(**3**)*

LiN=C*t*Bu_2_ (161 mg, 1.1 mmol) was added in one portion to a solution of (Me_3_SiC_5_H_4_)TiCl_3_ (291 mg, 1.0 mmol) [27] in 20 mL of toluene precooled at −30 °C. The reaction mixture was warmed slowly to room temperature and was stirred overnight. The mixture was then filtered through a celite pad, and the filter cake was washed with toluene. The combined filtrate and wash were taken to dryness under reduced pressure to give a purple solid. The solid was then dissolved in a minimum amount of toluene layered with a small amount of hexane. The chilled (−30 °C) solution gave purple microcrystals (yield: 88%). ^1^H-NMR (500 MHz, CDCl_3_): δ 6.67 (t, 2H, *J* = 2.5 Hz, Cp-*H*), 6.63 (t, 2H, *J* = 2.5 Hz, Cp-*H*), 1.30 (s, 18H, -C(C*H*_3_)_3_), 0.36 (s, 9H, -Si(C*H*_3_)_3_). ^13^C{^1^H} NMR (125 MHz, CDCl_3_): δ 204.8 (N=*C*-*t*Bu_2_), 133.9 (Cp ring C1), 123.6, 119.4 (Cp ring C2-C5), 47.0 (-*C*(CH_3_)_3_), 30.4 (-C(*C*H_3_)_3_), -0.30 (-Si(*C*H_3_)_3_). EA Calcd. for C_17_H_31_Cl_2_NSiTi: C 51.52%, H 7.89%, N 3.53%. Found. C 51.64%, H 7.88%, N 3.49%.

### 2.4. Polymerization Procedure

In a typical homopolymerization reaction, a 250 mL Büchi (Uster, Switzerland) stainless-steel autoclave, equipped with a mechanical stirrer and with an external thermostatic bath connected to a temperature control unit for temperature regulation, was evacuated for 120 min at 80 °C and conditioned three times with nitrogen. After cooling down to room temperature, the reactor was filled with toluene and with a solution of 25 mmol of MAO in toluene, which was previously prepared. After the thermal equilibration of the reactor system at the proper temperature, propylene was continuously added until saturation to the desired pressure. The reaction was initiated by injection of the precatalyst dissolved in toluene. The solution was stirred for 15 min, and the propylene pressure was kept constant during the polymerization reaction. The polymerization was terminated by the addition of a small amount of ethanol and hydrochloric acid and the polymer was precipitated upon pouring in ethanol, to which concentrated HCl had been added. The polymer was collected by filtration and dried under a vacuum at 70 °C until a constant weight was reached.

## 3. Results and Discussion

Our exploration of half-titanocenes Cp’TiCl_2_(N=C*t*Bu_2_) selected for the synthesis of propylene homopolymers included polymerization reactions by catalyst precursors, displayed in Scheme 1, in the presence of MAO, the analysis of polymer microstructure and chain end groups, molar masses and polymer properties. 

### 3.1. Polymerizaton

Propylene homopolymerizations were conducted in a 250 mL steel reactor using 10 µmol of catalyst and 25 mmol of MAO in 100 mL of toluene for 15 min with MAO ([MAO]/[Ti] = 2500) acting as a co-catalyst and scavenger, at two polymerization temperatures (40 and 60 °C) and propylene pressures (2 and 4 bar). The polymerization time was set at 15 min. Polymers were characterized by ^1^H- and ^13^C-NMR, DSC and SEC measurements. The results regarding the synthesis and characterization of selected polymers are summarized in Table 1. 

A comparison of the entries obtained at 2 and 4 bar at 40 °C evidences that polymerization activities with all catalytic systems increase with propylene pressure for propylene polymerization. A comparison of the entries at 40 and 60 °C shows that catalytic activities increase with temperature only with the permethylated catalyst **2**, while, with catalysts **1** and **3**, a decrease in activity is observed. This decrease is quite consistent with catalyst **3**. In general, polymer molar masses increase with monomer concentration (from 2 to 4 bar)—see entries 1 vs. 2 and 4 vs. 5—and molar mass distribution is narrow at 4 bar and 40 °C. The molar mass distribution of polymers obtained from catalysts **1** and **3** becomes bimodal at 60 °C (Figures S1–S3). This is probably related to the decrease in activity with catalysts **1** and **3**. Indeed, although these catalysts are generally stable at 60 °C [14], in the conditions of this study, a partial formation of different active species may occur, probably due to some remaining AlMe_3_ in dried MAO. The permethylation of the Cp ligand of catalyst **2** protects the catalyst from deactivation or active site changes; this causes greater activities by raising the temperature from 40 to 60 °C. Thus, for all the catalysts, the best results are obtained at 40 °C and 4 bar. 

All samples are soft solids, elastomeric materials, soluble in hydrocarbons and chlorinated solvents. DSC analysis showed no melting events, in agreement with data from the literature that report that moderately syndiotactic polypropylenes, with *rrrr* contents of about 20–25%, are completely amorphous [28]. The observed glass transitions are in a typical range for polypropylene materials with *T*_g_ values in the range of −2 to 2 °C, where the lower values are those of samples prepared at higher temperatures. 

### 3.2. Microstructure

**Figure 1:**
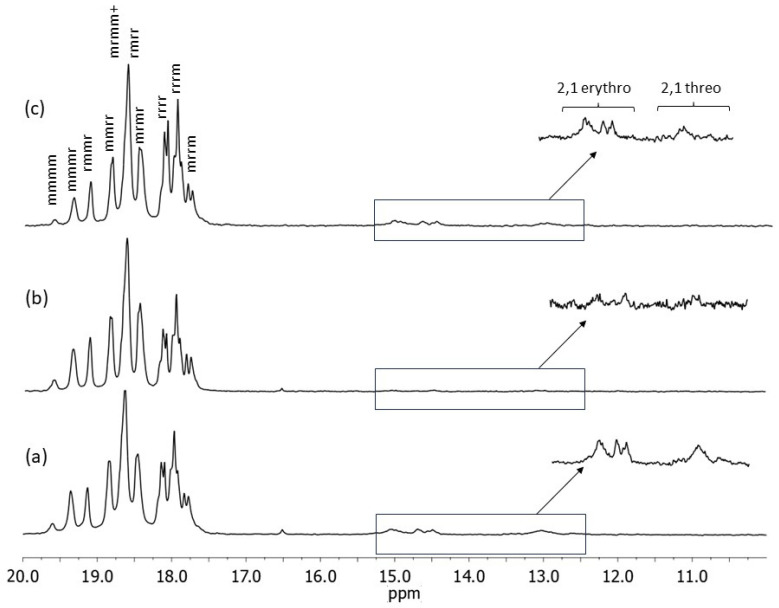
Expanded region of ^13^C-NMR spectra (108.58 MHz, C_2_D_2_Cl_4_, 103 °C) of polypropylene prepared at 40 °C and 4 bar by catalysts: **1**, entry 2 (**a**), **2** entry 5 (**b**) and **3** entry 8 (**c**). Peak labels refer to the pentad assignments reported in [29]. In the expanded region, regioirregular sequences are shown [30].

The microstructures of all the polypropylenes obtained with the three above mentioned catalyst systems were investigated by ^13^C-NMR spectroscopy (see Figure S4). In Figure 1, the methyl region of the ^13^C-NMR spectra of PP, obtained at 40 °C and 4 bar, from the three catalysts is presented. This region of the spectra shows the signals revealing the presence of all the pentads, indicating that all the three catalysts produce nearly atactic polypropylenes. The percentage of all the pentads of all the polymers obtained with the three catalysts, along with triads, the Bernoullian index [9] and regioirregularities, are displayed in Table S1.

All the polymers are slightly syndiotactoid; the *rrrr* and the ratio between *rrrr*/*mmmm* follow the order **1** > **3** > **2**. The Bernoullian factor B is below 1 for all polymers and tends to increase with temperature.

PPs produced by catalyst **2** are highly regioregular, those by catalyst **1** and **3** contain small amounts of both erythro and threo 2,1 misinsertions, as visible in the inserts of Figure 1a,c. They are around 5 mol% in the runs at 60 °C and 4 bar. 

**Scheme 2:**
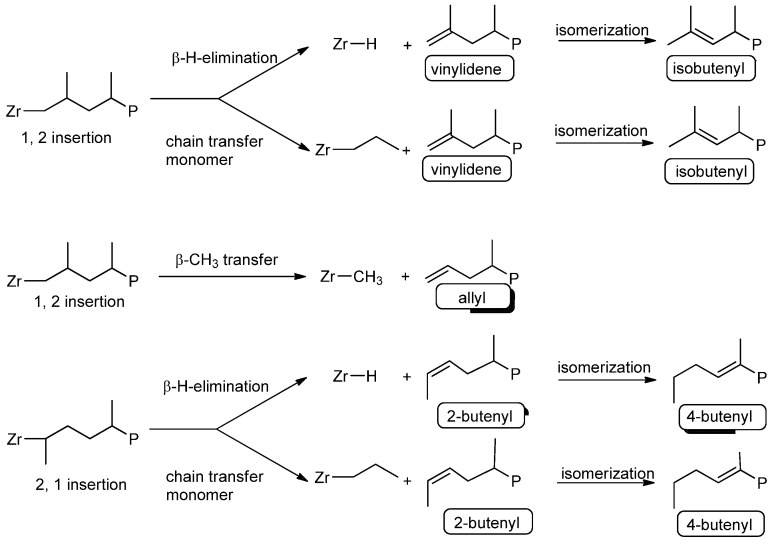
Unsaturated chain end groups in polypropylene.

Scheme 2 displays the typical termination processes in propylene polymerization, which lead to the creation of the unsaturated end groups. 

^1^H-NMR end-group analysis can give information about the chain transfer mechanisms operating with a certain catalyst. The expansions of the ^1^H-NMR spectra of polypropylenes obtained at 40 °C and 4 bar by catalyst **1**, **2** and **3** are displayed in Figure S5. The signal-to-noise ratio is quite high due to the high molar mass of the polymers. However, only signals of chain end groups formed after 1,2 insertions are visible.

**Figure 2:**
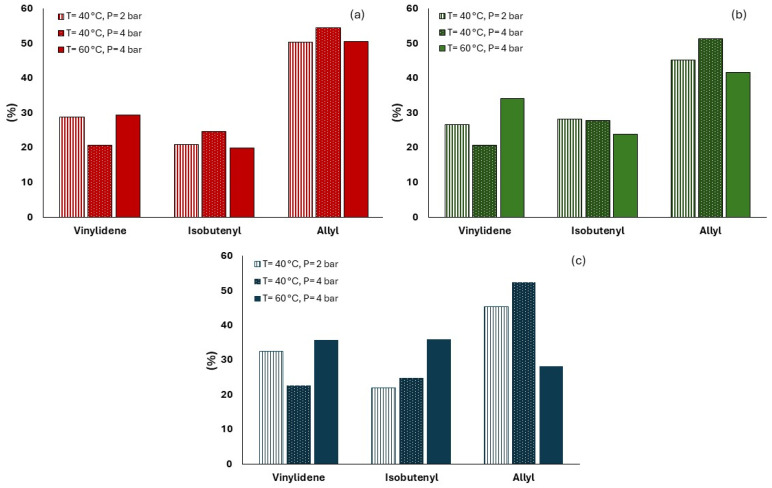
Chain end group trend as a function of temperature and pressure for catalyst **1** (**a**), catalyst **2** (**b**) and catalyst **3** (**c**).

The percentage of vinylidene, isobutenyl, and allyl end groups of PP samples obtained with the three catalysts are listed in Figure 2. Allyl chain end groups arising from β-methyl transfer increase with monomer concentration and decrease with the temperature. Vinylidene chain end groups, indicating the β-hydrogen elimination and/or chain transfer to monomer, show the opposite trend. The sum of vinylidene plus isobutenyl end groups is, in general, similar to the amount of allyl chain end groups. The relative decrease in allyl groups with temperature indicates an increase in β-hydrogen elimination in agreement with the decrease in molar masses with temperature. However, differences are not significant enough to give an indication of the differences in the polymerization mechanism. 

### 3.3. Mechanical Properties and Recyclibility

**Figure 3:**
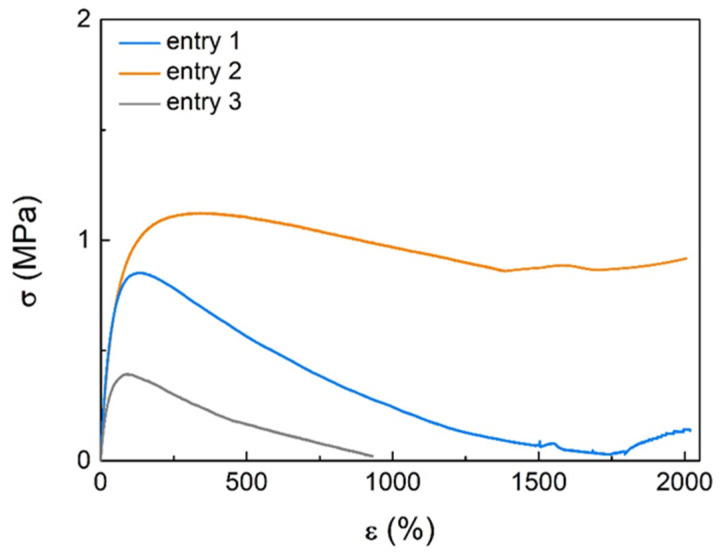
Stress–strain curve in the uniaxial tensile test of polypropylenes from catalyst **1**.

To evaluate the influence of molar masses and microstructure on the mechanical properties, uniaxial tensile and cyclic stress–strain tests were performed as well. The stress−strain curves of the investigated PPs generally first exhibit a linear elastic behavior and, subsequently, a progressive stress decrease with increasing strain, whereas no yielding and strain-hardening were observed. The stress–strain curves in the uniaxial tensile test of polypropylenes from catalyst **1** are reported in Figure 3 as an example. 

In Table 2, the Young’s modulus (*E*) was reported for all the samples and was found to be around 2.0–3.0 MPa for samples from catalysts **1** and **2**. Slightly higher values have been observed for entries 7 and 8 prepared by catalyst **3**. The maximum tensile strength (***σ_max_***) values were in the range of 0.3 to 1.2 MPa (Table 2). As already mentioned for Young’s modulus, ***σ_max_*** reached higher values for entries 7 and 8. The break features could not be determined for all samples; indeed, the elongation at break (*ε*) was higher than could be measured (>2000%) for all the samples prepared at 40 °C and 4 bar, independently from the catalyst, and for entries 1 and 7 prepared at 2 bar. This suggests tough, highly ductile materials. The tensile behavior is similar to that described by O’Hare et al. for fully amorphous ultra-high molar mass atactic PP (*M*_w_ up to 2.0 MDa and a low amount of 2,1-misinsertions) prepared in the presence of *ansa*-permethylindenyl-phenoxy titanium complexes immobilized on solid MAO [22]. The PP samples prepared at 60 °C and characterized by lower molar masses exhibited *ε* values spanning from 500 to 1000%.

**Figure 4:**
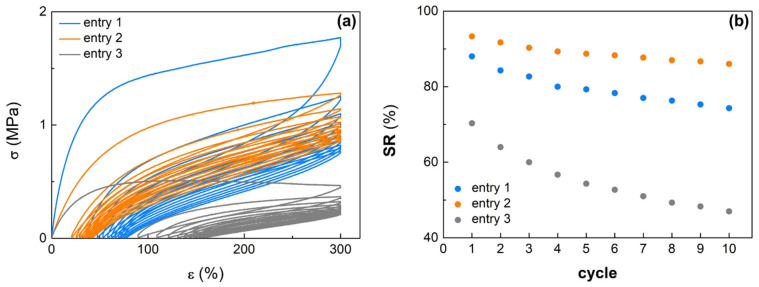
(**a**) Stress–strain curve in the hysteresis test for ten cycles at 300% strain; (**b**) strain recovery as a function of cycle number at 300% strain of polypropylenes from catalyst **1**.

The elastic recovery is the aptitude of an elastomer to restore its original shape and size after the removal of the force and is a crucial factor in assessing its properties. Thus, the samples obtained by catalyst **1** were continuously cycled 10 times at 300% strain, and the elastic recovery was evaluated after the removal of the strain for all cycles (Figure 4 and Table S3).

The highest extent of irreversible deformation occurs in the first cycle; after that, a slight enhancement in the unrecovered strain takes places in following cycles (Figure 4a). Entries 1 and 2 exhibit an excellent elastic recovery over the entire cyclic test, that is, 93 and 88% after the first cycle, and 86 and 75% after being cyclically stretched at 300% strain 10 times, respectively (Figure 4b and Table S3). Conversely, entry 3 shows a high extent of irreversible deformation after the first cycle with SR equal to 70% and low elastic recovery (47%) at the end of the last cycle (Figure 4b and Table S3). 

These outstanding mechanical properties are justified assuming that, since the samples possess an amorphous nature, the elastic network derives from entanglements caused by the ultra-high molar masses, which restrict permanent deformation by decreasing the mobility of the chains [22,31]. In detail, entanglements, defined as physical constraints to macromolecular dynamics in the amorphous regions, influence the mechanical behavior of a polymer system. Thus, ultra-high molecular mass polymers, having a high density of entanglements due to their long chain lengths, present a higher elasticity with respect to lower molecular mass polymers. Indeed, entries 1 and 2, obtained by catalyst **1**, and entries 7 and 8, obtained by catalyst **3**, possess *M*_w_ higher than 1000 Kg/mol and exhibit better mechanical performances in terms of elongation at break and strain recovery.

**Figure 5:**
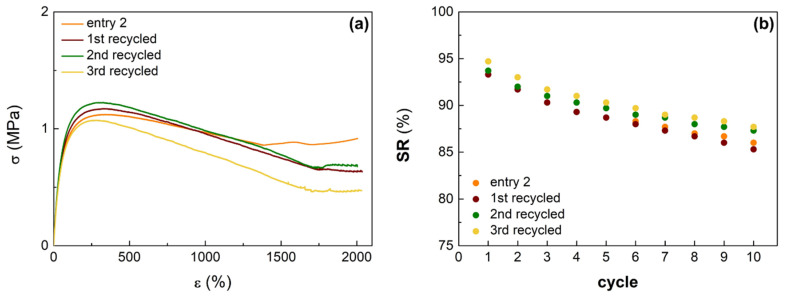
(**a**) Stress–strain curve in tensile test and (**b**) strain recovery as a function of cycle number at 300% strain in the hysteresis experiment for the pristine and recycled entry 2 samples.

The reprocessability of entry 2, that is, the polypropylene with the best tensile properties, was assessed by repeatedly melting and molding the fractured specimens at the processing conditions of the initial preparation, namely in a press at 180 °C and 50 bar for 5 min, thus obtaining recycled films with a 200 μm thickness. To evaluate the recyclability of entry 2, the reprocessing test was replicated three times, and the reshaped specimens underwent uniaxial tensile and 300% strain cycle tests. The stress–strain diagrams and mechanical properties of the pristine and reprocessed polypropylenes from entry 2 are shown in Figure 5a and Table S4, respectively. The stress–strain curves of the reprocessed specimens nearly overlap with the original one; therefore, even after the third reprocessing cycle, the mechanical properties of entry 2 change marginally.

Notably, after being recycled three times, entry 2 still showed great elasticity with strain recoveries similar to those of pristine samples (Figure 5b and Table S5).

## 4. Conclusions

Propylene polymerization by ketimide-modified half-titanocene catalysts, Cp’TiCl_2_(N=C*t*Bu_2_), in the presence of MAO has been studied. These catalysts were demonstrated to be highly active for propylene polymerization, especially at 4 bar and 40 °C. The Cp*-ketimide, C_5_Me_5_TiCl_2_(N=C*t*Bu_2_) (**2**), exhibited higher catalytic activities, while Cp’TiCl_2_(N=C*t*Bu_2_) (**1**,**3**) yielded very high molecular mass polymers, *M*_w_ up to 1400 Kg/mol. ^13^C-NMR analysis of the polymer microstructure showed that all the synthesized polypropylenes are atactic and highly regioregular, with predominant *rrrr* pentads, especially with catalyst **1**. Fully amorphous aPP, with no melting endotherm events and glass transition temperatures between −2 and 2 °C, were observed by DSC. These aPP, thanks to their high molar mass, behave like high-performance thermoplastic elastomers exhibiting exceptionally high ductility, and a tensile strain at break higher than 2000%. It is worth emphasizing that these materials maintain outstanding mechanical properties, namely high elongation and strain recovery, even after being molded and recycled several times.

Thus, for the first time, it was demonstrated that ketimide-modified half-titanocene catalysts selected give a high yield of aUHMWPP, with exceptional tensile and elastic properties, similar to commercially engineered thermoplastic elastomers.

## Figures and Tables

**Table 1:** Selected results and conditions for propylene polymerization with catalysts **1**–**3**.^1^

Entry	Catalyst	*T* (°C)	*P* (bar)	Yield (g)	Activity (Kg/(mol-Ti·h)	*M*_w_ ^2^ (Kg/mol)	*Ð* ^2^	*T_g_* ^3^ (°C)
1	**1**	40	2	4.06	1626	1250	2.7	1
2		40	4	6.08	2433	1404	1.7	1
3		60	4	3.85	1543	663	7.3	-2
4	**2**	40	2	1.59	627	660	1.8	2
5		40	4	11.20	4480	832	2.6	2
6		60	4	12.93	5173	650	1.7	0
7	**3**	40	2	4.66	1862	1303	4.0	0
8		40	4	9.16	3663	1240	1.5	1
9		60	4	1.90	761	627	3.7	-2

^1^ Reaction conditions: solvent= toluene, total volume of solvent = 100 mL; catalyst = 10 µmol, MAO = 25 mmol, *T* = 15 min. ^2^ Determined by SEC in *o*-dichlorobenzene with standard polystyrene calibration. ^3^ From DSC analysis.

**Table 2:** Tensile mechanical properties of polymers described in Table 1.

Entry	Catalyst	*E* (MPa)	*σ_max_* (MPa)	*ε* (%)
1	**1**	3.2 ± 0.3	0.91 ± 0.08	>2000
2		2.5 ± 0.3	1.05 ± 0.10	>2000
3		2.3 ± 0.1	0.39 ± 0.01	915 ± 70
4	**2**	1.9 ± 0.1	0.70 ± 0.06	790 ± 99
5		3.1 ± 0.3	0.78 ± 0.03	>2000
6		2.0 ± 0.3	0.56 ± 0.05	421 ± 23
7	**3**	6.0 ± 0.4	1.11 ± 0.01	>2000
8		4.1 ± 0.1	1.24 ± 0.08	>2000
9		1.3 ± 0.1	0.31 ± 0.02	483 ± 45

## Data Availability

Data is contained within the article or Supplementary Material.

## Supplementary Materials

The following are available online at https://www.mdpi.com/article/10.3390/polym16040512/s1: Figure S1: SEC chromatograms of entries 1, 2 and 3 from catalyst 1 (Table 1), Figure S2: SEC chromatograms of entries 4, 5 and 6 from catalyst 2 (Table 1), Figure S3: SEC chromatograms of entries 7, 8 and 9 from catalyst 3 (Table 1), Figure S4: ^13^C-NMR spectra (108.58 MHz, C_2_D_2_Cl_4_, 103 °C) of polypropylene samples prepared at 40 °C and 4 bar by catalyst 1 (a), catalyst 2 (b), and catalyst 3 (c), Figure S5: Olefinic region expansion of ^1^H-NMR spectra (108.58 MHz, C_2_D_2_Cl_4_, 103 °C) of PP samples prepared at 40 °C and 4 bar by catalyst 1(a); catalyst 2 (b); and catalyst 3 (c), Table S1: ^13^C and ^1^H-NMR characterization of polypropylenes prepared with catalysts 1–3 and MAO, Table S2: Unsaturated chain end groups observed by ^1^H-NMR of polypropylenes, Table S3: Strain recovery of polypropylenes from catalyst 1, Table S4: Tensile mechanical properties for the pristine and recycled entry 2 samples, Table S5: Strain recovery for the pristine and recycled entry 2 samples.
