# Supplementary material for: Amorphous Elastomeric Ultra-High Molar Mass Polypropylene in High Yield by Half-Titanocene Catalysts
Source: Polymers (Basel). 2024 Feb 14;16(4):512. doi: 10.3390/polym16040512 (PMC10893264; doi:10.3390/polym16040512)
Supplement: Supplementary file 1 [file polymers-16-00512-s001.zip › polymers-2846043-supplementary.pdf]

polymers

MDPI

Amorphous elastomeric ultra-high molar mass polypropylene in high yield by half-titanocene catalysts

Simona Losio, Fabio Bertini, Adriano Vignali, Taiga Fujiokka, Kotohiro Nomura and Incoronata Tritto

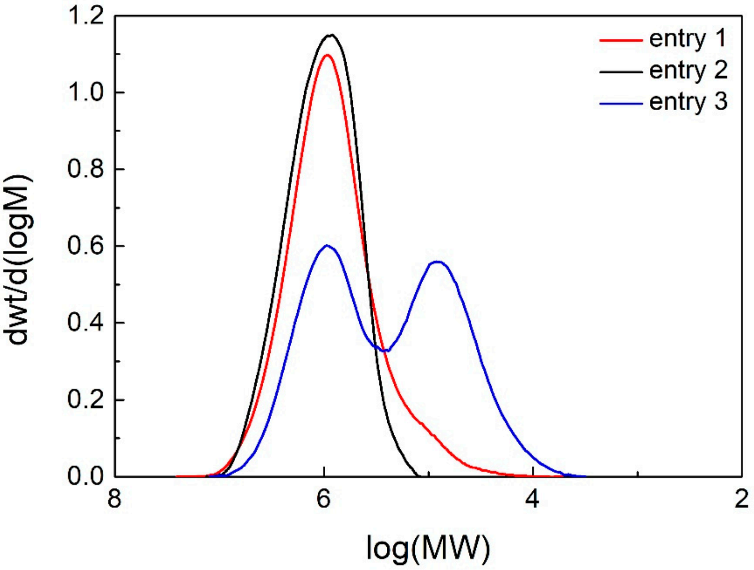

Figure S1. SEC chromatograms of entries 1, 2 and 3 from catalyst **1** (Table 1).

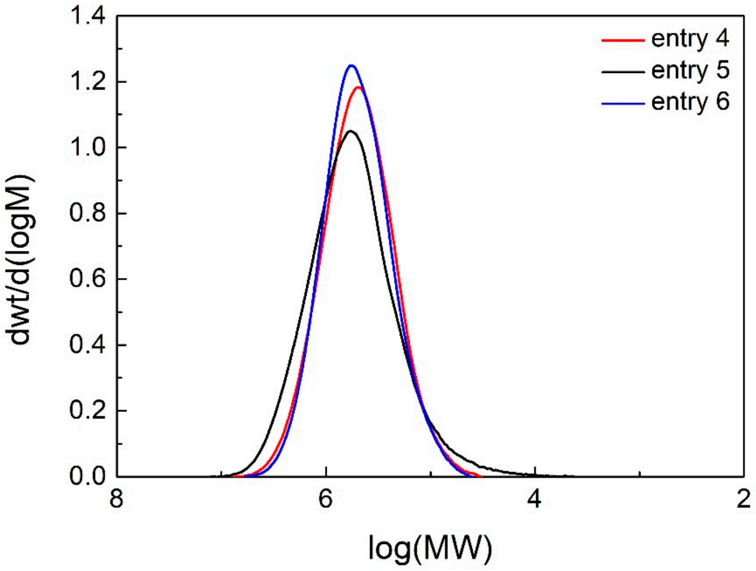

Figure S2. SEC chromatograms of entries 4, 5 and 6 from catalyst **2** (Table 1).

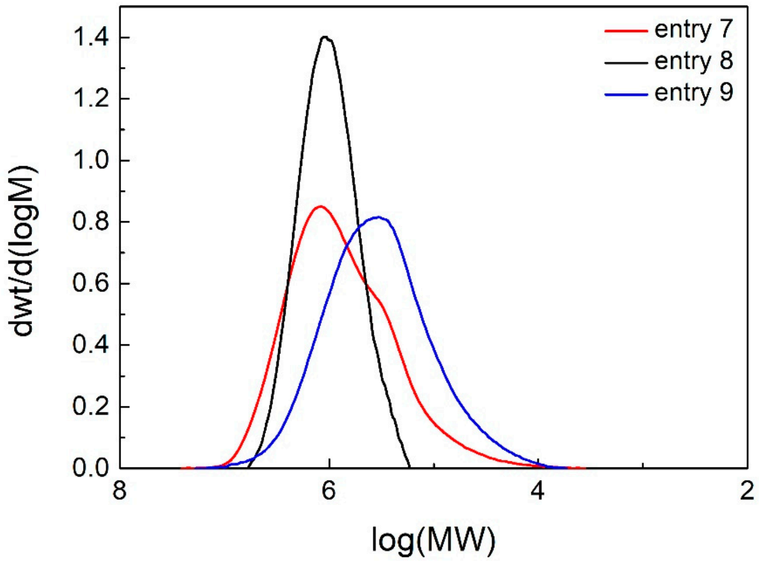

Figure S3. SEC chromatograms of entries 7, 8 and 9 from catalyst **3** (Table 1).

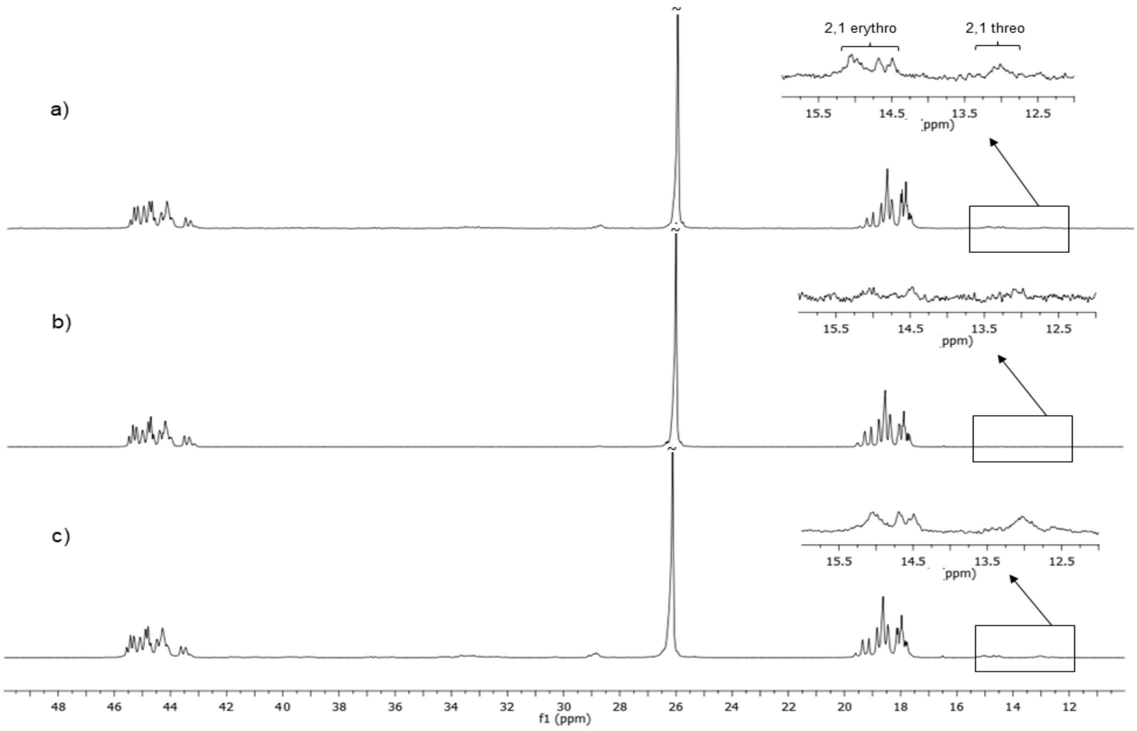

Figure S4. ^{13}C-NMR spectra (108.58 MHz, $C_2D_2Cl_4$, 103 °C) of polypropylene samples prepared at 40 °C and 4 bar by: catalyst **1** (**a**), catalyst **2** (**b**), and catalyst **3** (**c**).

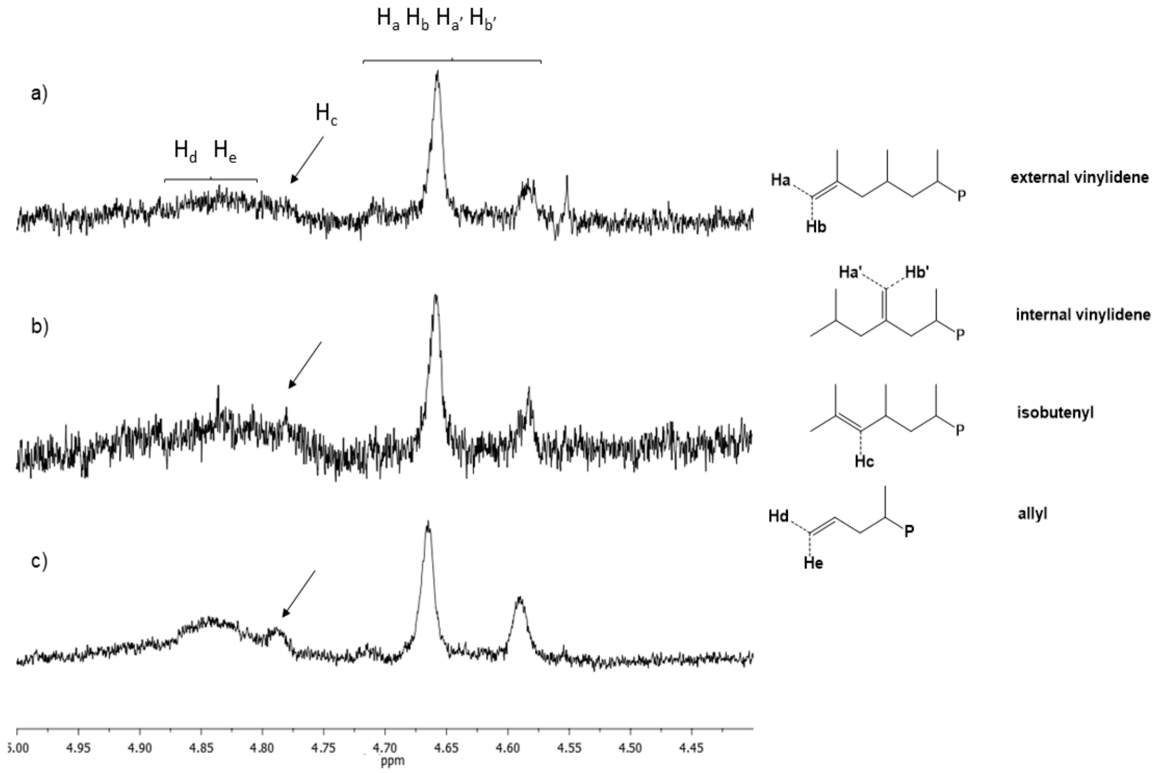

Figure S5. Olefinic region expansion of ^{1}H-NMR spectra (108.58 MHz, $C_2D_2Cl_4$, 103 °C) of PP samples prepared at 40 °C and 4 bars by: catalyst **1** (a); catalyst **2** (b); and catalyst **3** (c).

Table S1. ^{13}C -NMR characterization of polypropylenes prepared with catalysts **1-3** and MAO[a]

	mm	mr	rr	mmmm	mmmr	rmmr	mmrr	mmrm +rmrr	mrmr	rrrr	rrrm	mrrm	Bernoullian index [b]	regioirregularities	
														2,1 erythro	2,1 threo
1	10.72	48.72	40.56	1.05	4.46	5.21	9.67	25.37	13.68	14.87	18.62	7.07	0.73	3.56	1.82
2	10.76	47.52	41.72	1.12	4.42	5.22	9.42	24.77	13.33	15.27	18.3	8.15	0.80	1.85	0.74
3	11.34	47.65	41.00	1.34	4.51	5.49	9.03	25.44	13.18	13.18	19.16	8.66	0.82	5.48	4.66
4	15.60	52.20	32.20	2.14	7.22	6.24	11.25	26.1	14.85	9.71	15.08	7.41	0.74	0.57	0.32
5	15.32	51.74	32.94	2.04	6.71	6.57	11.12	25.93	14.69	10.63	15.2	7.11	0.75	0.71	0.32
6	16.03	49.96	34.01	1.77	8.13	6.13	10.6	24.99	14.37	10.97	15.81	7.23	0.87	0.61	0.77
7	13.65	49.41	36.93	1.74	6.05	5.86	10.33	25.96	13.12	11.31	17.31	8.31	0.83	3.58	3.61
8	14.14	50.99	34.87	2.07	6.18	5.89	10.72	24.18	16.09	11.09	15.86	7.92	0.76	3.46	1.81
9	13.38	48.06	38.57	1.73	6.04	5.61	9.49	25.73	12.84	12.46	19.26	6.85	0.89	4.81	6.06

[a]Calculated from integrals related to pentad and region-error signals [32,33]

[b]Bernoullian index B = 4[mm][rr]/[mr]^2

Table S2. Unsaturated chain end groups observed by ^{1}H-NMR of polypropylenes.

Entry	catalyst	*T* (°C)	*P* (bar)	Chain end group (%)		
				**Vinylidene**	**Isobutenyl**	**Allyl**
1	1	40	2	28.75	20.94	50.31
2		40	4	20.78	24.66	54.56
3		60	4	29.41	20.00	50.59
4	2	40	2	26.57	28.24	45.19
5		40	4	20.76	27.81	51.43
6		60	4	34.11	23.96	41.93
7	3	40	2	32.47	22.08	45.45
8		40	4	22.72	24.95	52.33
9		60	4	35.81	35.99	28.20

Table S3. Strain recovery of polypropylenes from catalyst **1.**

	entry		
	1	2	3
**Cycle**	**SR (%)**		
1	88	93	70
2	84	92	64
3	83	90	60
4	80	89	57
5	79	89	54
6	78	88	53
7	77	88	51
8	76	87	49
9	75	87	48
10	74	86	47

Table S4. Tensile mechanical properties for the pristine and recycled entry 2 samples.

Sample	*E* (MPa)	σ_{max} (MPa)	ε (%)
entry 2	2.5 ± 0.3	1.05 ± 0.10	> 2000
entry 2 - 1st recycled	2.6 ± 0.2	1.14 ± 0.05	> 2000
entry 2 - 2nd recycled	2.6 ± 0.2	1.15 ± 0.11	> 2000
entry 2 - 3rd recycled	2.4 ± 0.2	1.04 ± 0.04	> 2000

Table S5. Strain recovery for the pristine and recycled entry 2 samples.

	Sample			
	entry 2	1st recycled	2nd recycled	3rd recycled
**Cycle**	**SR (%)**			
1	93	93	94	94
2	92	92	92	93
3	90	90	91	92
4	89	89	90	91
5	89	89	90	90
6	88	88	89	90
7	88	87	89	89
8	87	87	88	89
9	87	86	88	88
10	86	85	87	88
